# Supplementary figures and images for: Spatial and temporal characteristics of surface soil moisture in a disturbed coal mining area of Chinese Loess Plateau
Source: PLoS One. 2022 May 4;17(5):e0265837. doi: 10.1371/journal.pone.0265837 (PMC9067677; doi:10.1371/journal.pone.0265837)

**S1 Fig. 24 days of Soil moisture data estimated based on SAR data**

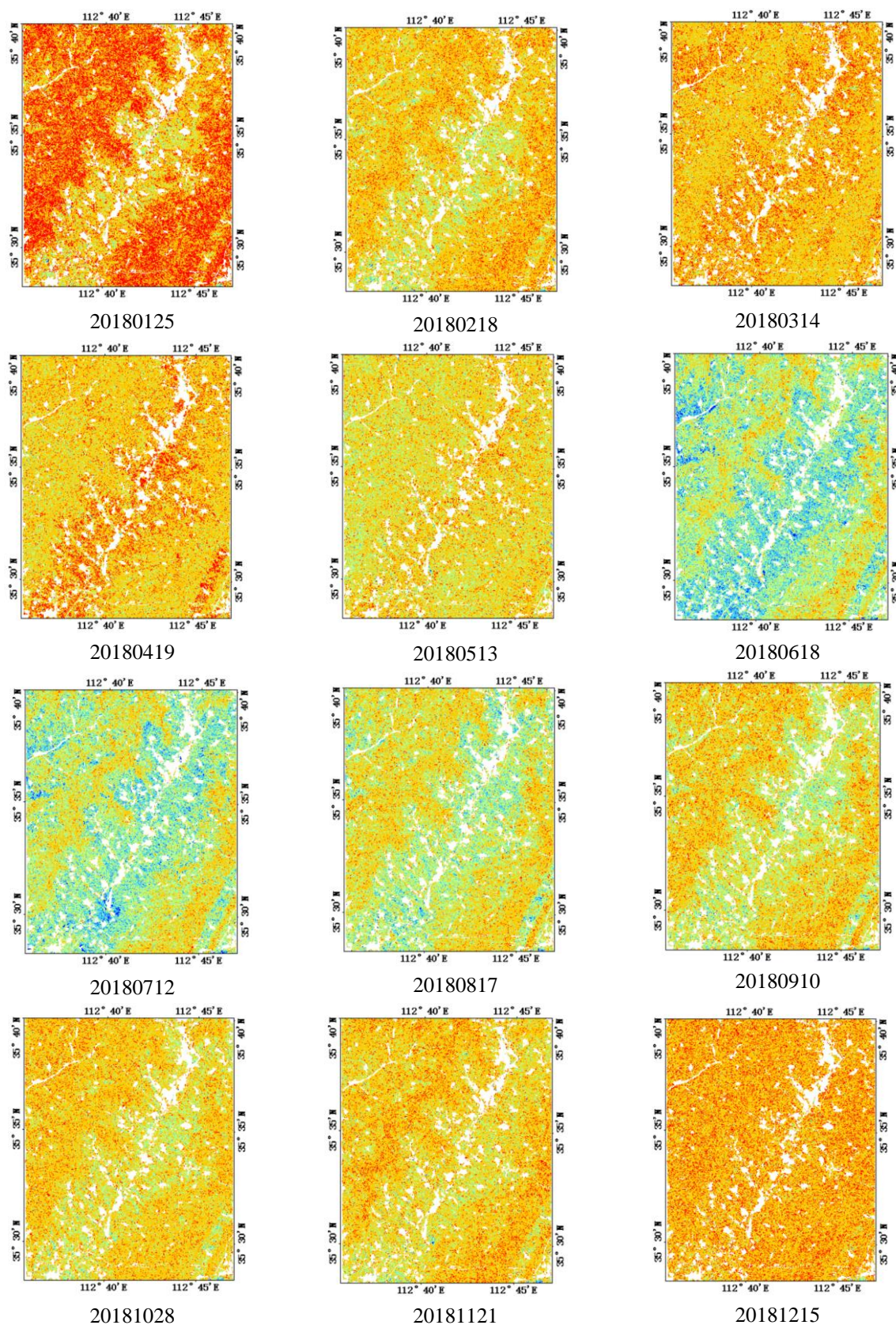

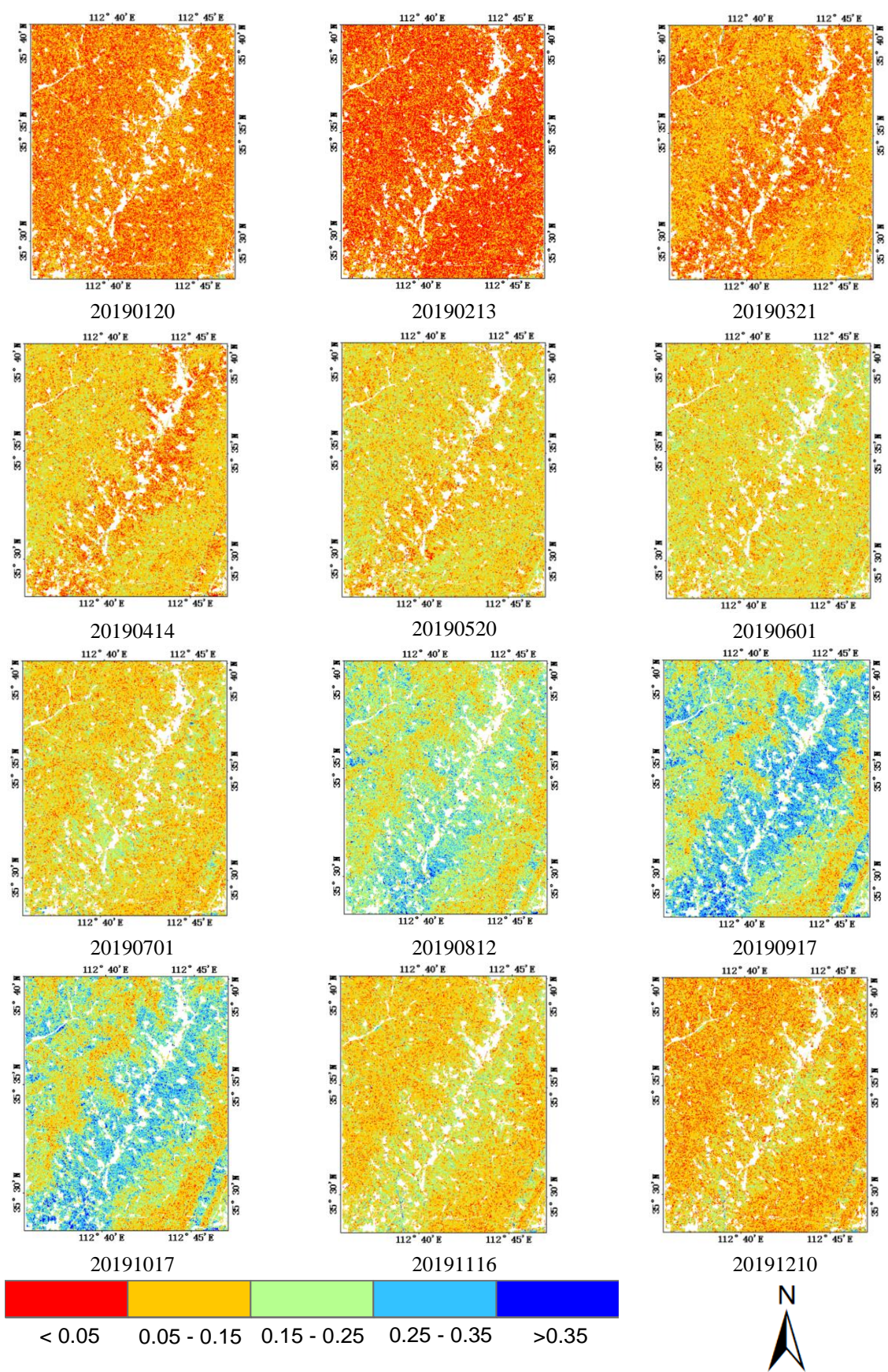

Supplement: S1 Fig — (PDF) [file pone.0265837.s001.pdf]

**S2 Fig. Data of soil texture and topographic characteristics in the study area**

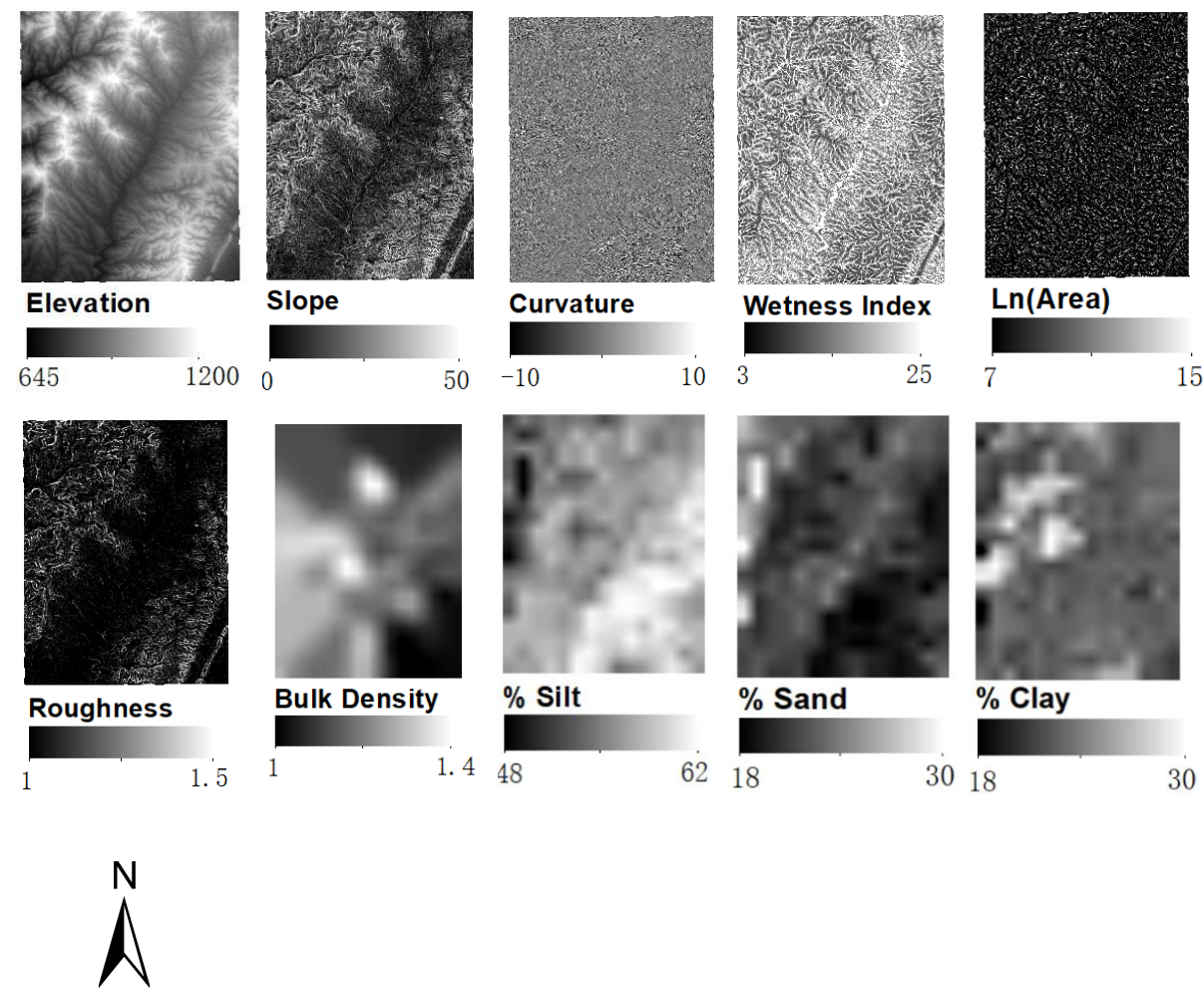

Supplement: S2 Fig — (PDF) [file pone.0265837.s002.pdf]
