# Supplementary material for: Spatial and temporal characteristics of surface soil moisture in a disturbed coal mining area of Chinese Loess Plateau
Source: PLoS One. 2022 May 4;17(5):e0265837. doi: 10.1371/journal.pone.0265837 (PMC9067677; doi:10.1371/journal.pone.0265837)
Supplement: Attachment — Submitted filename: comments to authors.docx [file pone.0265837.s003.docx]

***General comments to authors:***

The manuscript " Spatial and temporal characteristics of surface soil moisture in a disturbed coal mining area of Chinese Loess Plateau" shows an important issue: Temporal and Spatial Changes of Soil Moisture in Coal Mining Areas.

In this study, the author selected the coal mining area in the Changhe River Basin of the Loess Plateau as the research object. Using Synthetic Aperture Radar (Synthetic Aperture Radar) data, compared and analyzed the temporal and spatial variation characteristics of surface soil moisture within and outside the disturbance zone. And use the orthogonal function analysis method to analyze the potential time and spatial change characteristics of surface soil water in the Changhe River Basin and determine the regional distribution and change of soil moisture in the coal mining disturbance area. The selection of the research area is representative and the method is scientific, which provides a theory for ecological restoration in the mining area.

However, unfortunately the authors have not fully developed some topics of the manuscript and therefore I have decided that the manuscript needs revision. Overall, I recommend clarifying some aspects: introduction (for example, small changes are needed); of the methods in order to allow results interpretation; and results and discussion (for example, to make the results of the work more clear).

What is the practical significance of the conclusion drawn by the author, and what guiding significance and clearness does it have for other regions in China?

These are the main problems I found in the manuscript, and I hope they may help the authors when reviewing their work. Also see the PDF as several comments have also been added to it.

The detailed suggestions follow below.

***Detailed comments to authors:***

**Abstract:**

**Point 1:**

The research period should be clearly stated.

**Point 2:**

And there is a little doubt. What is the basis for selecting these 24 days, whether they are representative, and whether they can scientifically explain the changes in soil moisture in the mining area. If they can be compared in parallel periods, the conclusion of the paper will be more rigorous.

**Introduction:**

**Point 3**

The introduction should be further integrated, layer by layer, to show the innovation and practical significance of this article. In addition, the references of the article are too old to highlight the frontier research issues. you can cite other papers around the world.

**Materials and Methods:**

**Point 4**

Supplement and explain the source of the data and the selection of indicators.

**Point 5**

In Figure 1, "West of the Changhe River is designated as the disturbed area, and the area not affected by coal mining to the east of the Changhe River is designated as the undisturbed area", it is recommended that this part should be explained in the methodology, and Clarify the basis for the selection of disturbance area and non-disturbance area.

**Point 6**

In Figure 2, "Each point corresponds to a single radar signal difference……", this part should be explained in the methodology, or supplementary explanation in the form of notes.

**Point 7**

Regarding the methodology of "EOF Calculation", as an important part of the article, it should be explained in detail.

**Results and Discussion:**

**Point 8**

In 3.2, what does traditional analysis mean? The topic should be clear and easy to understand. Should also explain the meaning of this part, contribution to the article?

**Point 9**

In 3.2, line 2 of paragraph two. Is the author sure that Figure 4 can produce such a result?

**Point 10**

In 3.2, line 2 of paragraph three. The sources of these data are specified in the methodology.

**Point 11**

In 3.4, it is recommended that in the analysis of the driving factors of different models of soil moisture, the topography, soil texture and other factors selected in the study should be explained in the methodology.

**Point 12**

Figure 7 is recommended to be modified in accordance with the standard drawing standards, consistent with other drawings in the paper.

**Point 13**

The variables involved in Table 5 and Figure 9 should be supplemented in the methodology, and abbreviations should be annotated.

**Point 14**

For the differences of different influencing factors in different regions, conduct in-depth discussion. Where is the specific significance, the discussion should be sublimation of the result.

**Point 15**

The figures and tables in the manuscript are not uniform, and the fonts, sizes, and line widths in the figures are not uniform. It is recommended that all figures and tables in the manuscript are drawn uniformly in accordance with the specifications.

**Conclusion:**

**Point 16**

By displaying the results of the manuscript, it is necessary to improve the discussion part of the article. What are the practical guiding significance of these results to the non-disturbance area?

**References:**

**Point 17**

The references in the manuscript are too old to reflect the scientific frontiers of this research and the innovativeness of the problems, and do not support the results and the scientific nature of the discussion. It is recommended that the author can refer to more references around the world.
