# Supplementary material for: Spatial and temporal characteristics of surface soil moisture in a disturbed coal mining area of Chinese Loess Plateau
Source: PLoS One. 2022 May 4;17(5):e0265837. doi: 10.1371/journal.pone.0265837 (PMC9067677; doi:10.1371/journal.pone.0265837)
Supplement: Attachment — Submitted filename: Response to Reviewers.docx [file pone.0265837.s005.docx]

Dear Editor,

Thank you very much for handling our manuscript and we appreciate all the comments from the reviewers and yourself on our manuscript (PONE-D-21-26837) entitled " Spatial and temporal characteristics of surface soil moisture in a disturbed coal mining area of Chinese Loess Plateau". Corrections have been made based on your recommendations, and the detailed response to each comment is provided below. We highlighted all the corrections within the revised manuscript and in this letter. We are looking forward to receiving your positive news soon.

Sincerely,

Yi Cao

Hongfen Zhu

Rutian Bi

Yaodong Jin

Comments from Reviewer 1：
1. “These factors have left imbalances in regional water resources, so it is particularly necessary to monitor changes in soil water content in the study area”. It lacks of logic between the two sentences.

Response: We deleted this statement and restated the purpose and significance of this study.

2. The deficiencies of former studies in science is not clear. What’s the contribution of this study to the science?

Response: The study area is located in the hilly area of the Loess Plateau with various topographic fluctuations. Traditional soil moisture monitoring sampling points are difficult to reveal the large-scale variation characteristics of the mining area, and remote sensing monitoring technology can effectively and timely obtain regional soil water information. At present, there are few studies on the temporal and spatial changes of soil surface moisture under the influence of mining subsidence.

3. What is special of the research area? Why the soil moisture in disturbed coal mining area is important?

Response: Revealing the change characteristics of soil water content and environmental driving mechanism after coal mining disturbance will have reference significance for disaster control and ecological environment restoration in mining areas, and provide reliable decision support and scientific basis for the coordinated development of hilly areas of the Loess Plateau.

4. Why the surface soil moisture is important, especially in 5 cm?

Response: Surface soil water directly affects the growth of surface vegetation. According to previous studies, the effective soil depth of soil water estimated by SAR data is 5cm. Therefore, in this study, the accuracy of SAR data is verified by the measured data of 5cm soil surface.

5. What’s the significance of this study for application or for science?

Response: In this paper, the change characteristics of soil water content in the study area were obtained to provide theoretical basis and data support for land reclamation and vegetation restoration.

6. Why the SAR data and CD algorithm are employed? Why they are necessary?

Response: The soil moisture inversion method based on change detection algorithm does not need to measure the parameters such as surface roughness and vegetation. It only needs long-time radar data and optical data to effectively remove the influence of surface roughness and vegetation

7. The purpose of this study should be elaborated and described clearly.

Response: According to 1-6 points, the introduction of this paper is restated.

8. Figure 2. Why the relationship between backscattering coefficient difference and NDVI was expressed by using linear equation? What is the R2 of the equation? I don’t think there is obvious relationship between them.

Response: We used quantile regression analysis. The relationship between backscattering coefficient difference and NDVI is linear in the statistical interval of the upper decile. Reference: Qi G , Mehrez Z , Maria E , et al. Synergetic use of Sentinel-1 and Sentinel-2 data for soil moisture mapping at 100 m resolution[J]. Sensors, 2017, 17(9):1966.

9. Figure 3: The Chinese words should be deleted.

Response: The Chinese words were deleted.

10. The verification and application of the results were very weak. The discussion should be improved very much.

Response: We added the discussion content and clarified the shortcomings of the research and the future research direction. Discussions added in 3.4：

Tiejun Wang (2017) found that the primary spatial structure of soil moisture was mostly correlated with soil texture in all the study regions, indicating the dominant role of soil in determining soil moisture spatial variability.

EOF2 was negatively correlated with altitude and slope, which also shows that the soil water distribution is more dynamic at the location with higher altitude and steeper slope. This result is consistent with previous studies. They found that the slope position is the largest contributor to the temporal variability of soil moisture. Steeper slopes are expected to promote drainage and produce higher temporal variability. Higher altitude areas may be more easily separated from stable water sources, such as rivers or regional aquifers, so the soil water change in this area is more dynamic.

By comparing the differences of EOF modes between the sub-regions, we try to explain how coal mining activities affect the temporal and spatial distribution of surface soil water. The correlations analysis showed that the response directions of the sub- regions to the characteristics of altitude, slope, soil texture and bulk density are obviously different. We believe that this is related to the change of the original soil structure by the land remediation measures on the damaged land.

As mentioned in the introduction, there were few studies on the temporal and spatial changes of soil surface water in mining areas of the Loess Plateau under the influence of mining subsidence. In this paper, we only focus on the correlation between soil type, topographic characteristics and soil water distribution model. However, vegetation cover also plays a very important role in the temporal and spatial distribution of surface soil water. For example, EOF1 values along both banks of the river and hillside areas were significantly different. The response directions of the two regions to time changes were opposite. On the one hand, it may be due to the horizontal redistribution of soil moisture on the date of more precipitation, on the other hand, it may be related to the planting of seasonal crops along both banks of the river, such as winter wheat. In contrast, the soil in the hillside area remains relatively dry on dry and wet days, because the soil covered by shrubs and trees will lose water due to transpiration even in wet periods. However, we lack long-term observed vegetation parameters in the study area, so there is no quantitative comparison of the impact of vegetation cover parameters on surface soil water variability in the study area. Future research will compare and analyze the soil water distribution mode by collecting long-term observed vegetation and hydrological parameters.

Comments from Reviewer 2：
Abstract:
Point 1:
The research period should be clearly stated.

Response: The research period was added: from Jan 25, 2018 to Dec 10, 2019

Point 2:
And there is a little doubt. What is the basis for selecting these 24 days, whether they are representative, and whether they can scientifically explain the changes in soil moisture in the mining area. If they can be compared in parallel periods, the conclusion of the paper will be more rigorous.

Response: Sentinel-1 radar data interval is 12 days. Sentinel-2 optical data were also used in this paper. Considering the influence of cloud cover during satellite transit on optical data, the data interval is 24 days or 36 days. The time span of this study is 2 years. Based on the data of these 2 years, we have made a methodological attempt. We will continue to conduct in-depth research. Thank you for your suggestions.

Introduction:
Point 3
The introduction should be further integrated, layer by layer, to show the innovation and practical significance of this article. In addition, the references of the article are too old to highlight the frontier research issues. you can cite other papers around the world.

Response: The research significance of this paper was restated. The latest relevant papers were reviewed.

Materials and Methods:
Point 4
Supplement and explain the source of the data and the selection of indicators.

Response: The source of the data and indicator selection were supplemented. All data can be checked in S2 Fig. and explanations were added in 2.3:

(4) Soil texture and topographic attributes

The digital elevation model (DEM) of the study area adopted ASTER GDEM. The factors such as slope, curvature, terrain roughness, natural logarithm of catchment area (Ln Area) and terrain wetness index (WTI), were extracted from the DEM of the study area. Soil texture such as silt, sand, clay and bulk density (BD) and precipitation data were obtained from the National Earth System Science Data Center (China).

Point 5
In Figure 1, "West of the Changhe River is designated as the disturbed area, and the area not affected by coal mining to the east of the Changhe River is designated as the undisturbed area", it is recommended that this part should be explained in the methodology, and Clarify the basis for the selection of disturbance area and non-disturbance area.

Response: The study area is divided into East and west areas by Changhe river. There are coal resources underground in the western region. Large area of surface damage is caused by coal mining. People have implemented large-scale land remediation on the damaged land, so we defined this region as a disturbed area. There is no coal resource underground in the eastern region, which we called the undisturbed area.

Point 6
In Figure 2, "Each point corresponds to a single radar signal difference……", this part should be explained in the methodology, or supplementary explanation in the form of notes.

Response: This sentence has been modified: Each point corresponds to the radar signal difference and NDVI in the same pixel position

Point 7
Regarding the methodology of "EOF Calculation", as an important part of the article, it should be explained in detail.

Response: A brief description of EOF calculation method was added:

For a soil moisture data set with n locations and m observations at each location, spatial anomalies of soil moisture can be computed as

${Z_{i}(t)=S}_{i}(t)-\frac{1}{m}\sum_{j=1}^{m} S_{j}(t)$	(10)

where Zi(t) and Si(t) are soil moisture spatial anomaly and volumetric soil moisture content at location i and time t, respectively. A matrix of soil moisture spatial anomalies, Z, can be constructed as

$Z=\left[ \begin{matrix} Z_{11} & \cdots& Z_{1n} \\ \vdots& \ddots& \vdots\\ Z_{m1} & \cdots& Z_{mn} \end{matrix} \right]$	(11)

Then, a covariance matrix V can be calculated as

$V=\frac{1}{m-1}Z^{T}Z$	(12)

where the superscript T indicates matrix transpose. To perform EOF analysis, one needs to find eigenvectors and eigenvalues for V, which satisfy the following equation:

$VE=LE$	(13)

where E contains eigenvectors in columns

$E=\left[ \begin{matrix} e_{11} & \cdots& e_{1n} \\ \vdots& \ddots& \vdots\\ e_{n1} & \cdots& e_{nn} \end{matrix} \right]$	(14)

and L contains eigenvalues along the diagonal

$L=\left[ \begin{matrix} l_{11} & \cdots& 0 \\ \vdots& \ddots& \vdots\\ 0 & \cdots& l_{nn} \end{matrix} \right]$	(15)

The above procedure rotates the original coordinate axes with each axis indicating a sampling time into a new set of orthogonal coordinate axes with each eigenvector representing a new axis. The eigenvalues explain the variance in the data along the direction of each corresponding new axis, and the portion of the explained variance (Pj) by the jth new axis in the total variance can be computed as

$P_{j}=\frac{l_{jj}}{\sum_{k=1}^{n} l_{jj}}$	(16)

The eigenvectors are then arranged according to eigenvalues: the first axis explains the largest variance in the data, while each following axis explains the largest remaining variance and is orthogonal to other axes. F can be computed by projecting Z onto E

$F=ZE$	(17)

The purpose of EOF analysis is to reduce the dimensionality of a data set, and the approach of North et al. adopted to select statistically significant EOFs. This approach assumes that an EOF is statistically significant if the lower confidence limit (e.g., 95%) of its eigenvalue is greater than the upper confidence limit of the remaining largest eigenvalue. The 95% confidence interval for the kth eigenvalue can be calculated as

${{CL}_{k}=\lambda}_{k}\times\left( 1\pm\sqrt{\frac{2}{m}} \right)$	(18)

where CIk is the confidence interval for the kth eigenvalue and m is the number of sampling locations.

Results and Discussion:
Point 8
In 3.2, what does traditional analysis mean? The topic should be clear and easy to understand. Should also explain the meaning of this part, contribution to the article?

Response: Compared with EOF analysis, we conducted traditional statistical analysis on the data of soil water in 24 days, such as maximum and minimum value, average value and variance. These analyses could show the periodic changes of soil water in 24 days with seasons. This part served as a supplement to EOF analysis.

Point 9
In 3.2, line 2 of paragraph two. Is the author sure that Figure 4 can produce such a result?

Response: A total of 24 periods of data were calculated in this paper, but due to space constraints, only two contrasting data were shown in this paper. We have added two periods of data to explain the relevant statements. All data can be checked in S1 Fig (Supporting information).

Point 10
In 3.2, line 2 of paragraph three. The sources of these data are specified in the methodology.

Response: The sources of data were described in materials and methods

Point 11
In 3.4, it is recommended that in the analysis of the driving factors of different models of soil moisture, the topography, soil texture and other factors selected in the study should be explained in the methodology.

Response: These data were described in materials and methods. All data can be checked in S2 Fig (Supporting information).

Point 12
Figure 7 is recommended to be modified in accordance with the standard drawing standards, consistent with other drawings in the paper.

Response: These errors have been modified

Point 13
The variables involved in Table 5 and Figure 9 should be supplemented in the methodology, and abbreviations should be annotated.

Response: These errors have been modified

Point 14
For the differences of different influencing factors in different regions, conduct in-depth discussion. Where is the specific significance, the discussion should be sublimation of the result.

Response: We added some content to the discussion section. We try to explain whether coal mining activities affect the temporal and spatial distribution of surface soil water.

Point 15
The figures and tables in the manuscript are not uniform, and the fonts, sizes, and line widths in the figures are not uniform. It is recommended that all figures and tables in the manuscript are drawn uniformly in accordance with the specifications.

Response: These errors have been modified

Conclusion:
Point 16
By displaying the results of the manuscript, it is necessary to improve the discussion part of the article. What are the practical guiding significance of these results to the non-disturbance area?

Response: We added the shortcomings of this study and the future research direction. The research results mainly focused on the impact of coal mining activities on the temporal and spatial distribution of surface soil water. The undisturbed area was only used for comparative analysis.

References:
Point 17
The references in the manuscript are too old to reflect the scientific frontiers of this research and the innovativeness of the problems, and do not support the results and the scientific nature of the discussion. It is recommended that the author can refer to more references around the world.

Response: We added the latest references related to this study
